# Subjective versus objective, polymer bur-based selective carious tissue removal: 1-year interim analysis of a randomized clinical trial

**DOI:** 10.1038/s41598-020-66074-x

**Published:** 2020-06-04

**Authors:** Marta Gomes Marques, Leandro Augusto Hilgert, Larissa Ribeiro Silva, Karine Medeiros Demarchi, Patrícia Magno dos Santos Matias, Ana Paula Dias Ribeiro, Soraya Coelho Leal, Sebastian Paris, Falk Schwendicke

**Affiliations:** 1 Graduate Program in Dentistry, University of Brasília, Brasília, Brazil; 2 Department of Dentistry, University of Brasília, Brasília, Brazil; 3 Graduate Program in Dentistry, University of Brasília, Brasília, Brazil; 4 Graduate Program in Health Sciences, University of Brasília, Brasília, Brazil; 5 Department of Restorative Dental Sciences, University of Florida College of Dentistry, Gainesville, Florida USA; 6 Department of Oral Diagnostics, Digital Health, Health Services Research, Charité– Universitätsmedizin, Berlin, Germany

**Keywords:** Clinical trial design, Health care, Dentistry

## Abstract

We aimed to compare subjective (S) vs. objective (O) selective carious tissue removal using hand-excavation versus a self-limiting polymer bur, respectively. A community-based single-blind cluster-randomized controlled superiority trial was performed. This is a 1-year-interim analysis. 115 children (age 7–8 years) with ≥1 vital primary molar with a deep dentin lesion (>1/2 dentin depth) were included (60 S/55 O). The cluster was the child, with eligible molars being treated identically (91 S/86 O). Cavities were prepared and carious tissue on pulpo-proximal walls selectively removed using hand instruments (S), or a self-limiting polymer bur (Polybur P1, Komet). Cavities were restored using glass-hybrid material (Equia Forte, GC). Treatment times and children’s satisfaction were recorded. Generalized-linear models (GLM) and multi-level Cox-regression analysis were applied. Initial treatment times were not significantly different between protocols (mean; 95%CI S: 433; 404–462 sec; O: 412; 382-441 sec; p = 0.378/GLM). There was no significant difference in patients’ satisfaction (p = 0.164). No pulpal exposures occurred. 113 children were re-examined. Failures occurred in 22/84 O-molars (26.2%) and 26/90 S-molars (28.9%). Pulpal complications occurred in 5(6%) O and 2(2.2%) S molars, respectively. Risk of failure was not significantly associated with the removal protocol, age, sex, dental arch or tooth type (p > 0.05/Cox), but was nearly 5-times higher in multi-surface than single-surface restorations (HR: 4.60; 95% CI: 1.70-12.4). Within the limitations of this interim analysis, there was no significant difference in treatment time, satisfaction and risk of failure between O and S.

## Introduction

For deep carious lesions, selective carious tissue removal, where soft tissue remains in pulpal cavity areas and is sealed beneath a (mainly adhesive) restoration is recommended over more invasive non-selective (“complete”) removal, mainly as the risk of pulp exposure is significantly reduced^1^. Alternatively, such lesions in primary molars may be managed via sealing them beneath stainless steel crowns (Hall Technique), if available^2^. Avoiding pulp exposure is relevant, as oftentimes more invasive therapies like pulpotomy or removal of the tooth are needed if the pulp is exposed in primary teeth. Some children, e.g. those with limited compliance and dental fear, may not accept these under local anesthetics. They may further come with the risk of systemic adverse events and high costs^1^.

For selective removal, the current standard technique is to subjectively (i.e. arbitrarily and not necessarily reproducible) remove carious dentin (using hand or rotary instruments) until only hard dentin remains peripherally, and soft, leathery or firm dentin in pulp-proximal areas. An alternative and more objective removal technique involves self-limiting polymer burs (like Polybur P1, Komet, Lemgo, Germany)^3^. These are manufactured from medical-grade polyether-ketone-ketone^4,5^, and are harder than soft dentin but softer than firm or hard dentin^6^, on which the bur abrades and hence does not remove any further hard tissue^7^.

Self-limiting burs have been validated *in vitro* for selectivity (removing less sound dentin than conventional carious tissue removal)^8^. Also, adhesive bond strengths to the dentin walls remaining after using these burs instead of conventional excavation have been tested, demonstrating decreased bond strengths when using self-limiting burs^9^. Clinically, though, such self-limiting burs have only sparsely been applied, mainly on their impact on peri-operative pain or the required removal time^10^. Hence, at present, it remains unclear if clinically relevant outcomes like the risk of pulp exposure or pulpal and restorative success (absence of complications) and tooth survival are improved if performing such “objective” instead of “subjective” selective removal^3,11,12^.

Therefore, the aim of this study was to compare subjective (S) versus objective (O) carious tissue removal of deep dentin lesions in primary molars with vital and non-symptomatic pulps, by means of a community-based cluster-randomized trial. Our hypothesis was that O is significantly more efficacious (higher success) than S. We here present a 1-year interim analysis.

## Methods

### Overview

**Figure 1:**
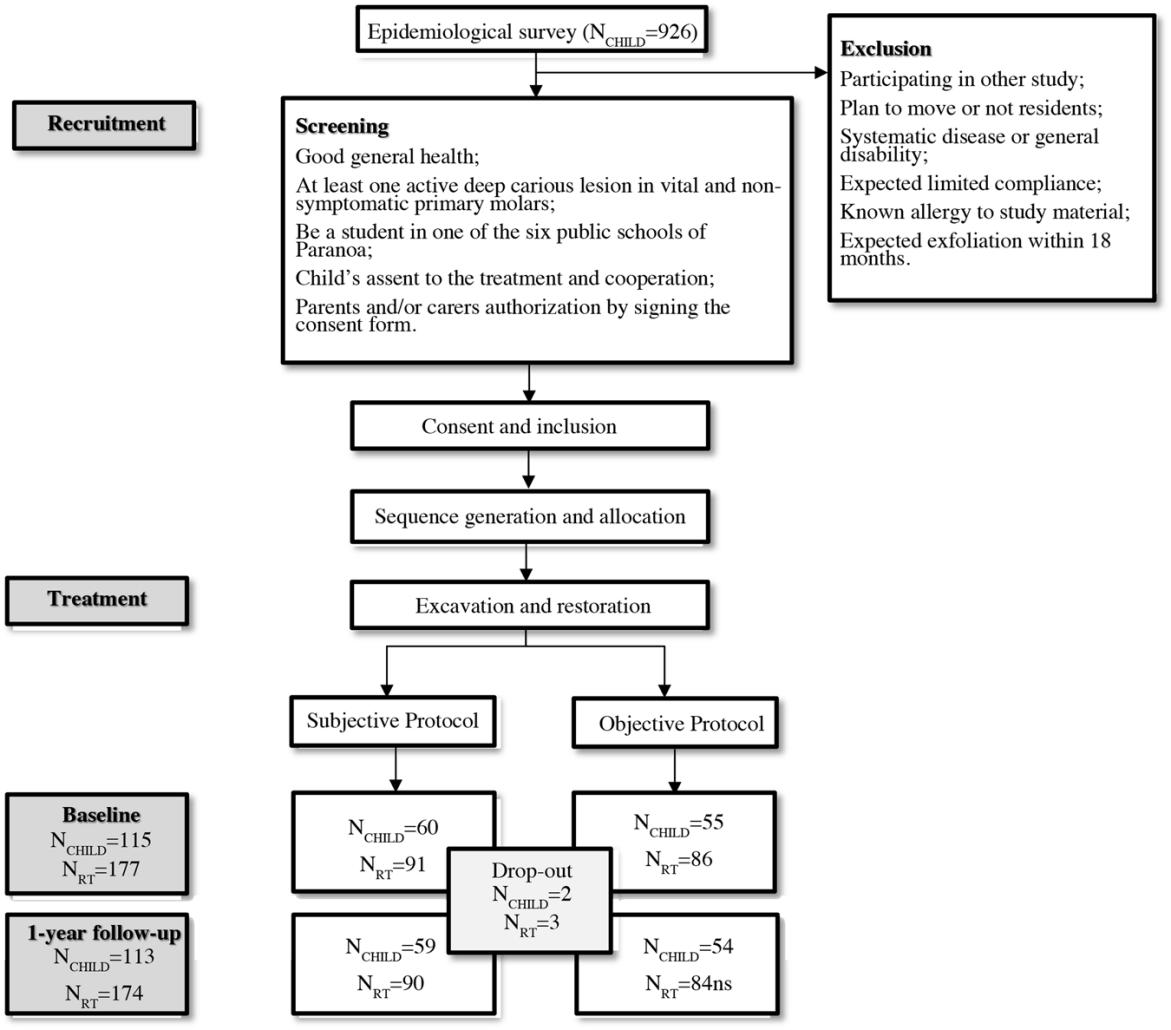
CONSORT flowchart. N_CHILD_ = number of children, N_RT_ = number of teeth restored.

This is a community-based single-blinded cluster-randomized controlled superiority trial. Reporting manuscript follows the CONSORT (Consolidated Standards of Reporting Trials) guidelines. The trial was approved by the Research Ethics Committee of the University of Brasília Medical School (1.400.687/2016) and registered at Clinicaltrials.gov (NCT02754466) 28.04.2016. We confirm that all research was performed in accordance with relevant guidelines (Declaration of Helsinki) and regulations. The protocol to this trial was published elsewhere^13^ and deviations from this protocol are laid out below. The CONSORT checklist is available within the supplementary files. The study flowchart is shown in Fig. 1.

### Sample size estimation

The unit of analysis was the tooth. Hence, this a multilayered cluster-randomized trial, with the patient and the school being the clusters. Clustering was taken into account using the so-called Lee, Wei and Amato (LWA) model for clustered survival data^14^. Sample size estimation for this model was based on the ideas of Xie and Waksman^15^. It was assumed that at 36 months (planned total follow-up time), 90% of O-molars and 80% of S-molars would show no complication (success). The assumption of superiority of the polymer bur protocol (O) was justified given the higher cost associated with this strategy and the ease of performing the conventional S protocol. Considering α = 0.05 and 1 − β = 0.9, as well as an intra-cluster correlation of 0.8 and a mean of 1.5 teeth treated per child, the required sample was 45 per group. Assuming an annual sample loss of 20%, the required sample was 57 per group. A total of 115 patients (177 teeth) participated in the study.

### Sample selection

This clinical trial is part of a larger study that aimed to evaluate the impact of oral health in children’s quality of life, anthropometric and cognitive development. Overall, 926 children were examined in six public schools of Paranoá, a deprived suburban area of Brasilia, the capital of Brazil. Given that children in this area of Brasilia come from a low socio-economic background and show a high mean d_3_mft at baseline, and considering this community having limited access to dental services^16^, we assume all children to show high caries risk. Note, though, that no formal caries risk assessment was provided in the present study.

All children received dietary advice and oral hygiene instructions. Two calibrated dentists performed the recruitment and treatment for this trial. The inclusion criteria were as follows: (1) age between 7–8 years; (2) good general health; (3) informed consent of the parents and/or carers (4) cooperation of the children for the required treatment steps; (5) at least one vital, clinically and radiographically non-symptomatic, retainable, deeply carious primary molar; and (6) the child being a student in one of the six schools of Paranoa (which was relevant to ensure follow-up).

The lesion needed to be active and to radiographically extend into the inner half of the dentin. An external examiner, previously calibrated, was responsible for measuring the depth of the lesions on radiographs. Single and multiple surface lesions were included; the size of the cavity (single or multiple surface) was recorded after carious tissue removal and preparation (see below).

Patients that were participating in another study or planning to move away, as well as those who were not residents of Paranoa, patients with systemic diseases or disabilities, with known allergies to dental materials used within the study, as well as those with expected limited compliance, and patients with teeth expected to exfoliate within the next 18 months were not included. Non-eligible patients were treated according to their need via referral to the pedodontics service of the University of Brasília.

Patients and their parents/carers were provided with information leaflets concerning the study and fully informed about the study verbally, too. Informed consent was obtained from all the parents/legally authorized representative of the participants included in the study when attending the second, treatment appointment in the mobile unit (see below). There were minimum 24 h and maximum one week between the first and second appointment.

### Sequence generation, allocation and blinding

A random sequence was generated via random numbers tables. The allocation was concealed using opaque sealed envelopes, with an overall 120 envelopes (60 per group) being used. Envelopes were only opened when the child was seated in the chair and ready for treatment. As 115 children were included on the study, study groups are not perfectly balanced (five envelopes were never opened).

Due the obvious differences between the restorative protocols, blinding of the operator and the patient was not possible. Nevertheless, since the restorative material was the same in both groups, examiners’ blinding during follow-up was feasible. In addition, the participants and their caregivers were informed about the importance of not providing information to the examiners about the study arm. Follow-up radiographs were not performed due to radiation protection reasons.

### Treatment procedure

Treatment was carried out between May and December 2017. During the treatment, data for each tooth and patient was collected using pilot-tested case report forms. We collected data on the treated tooth (dental arch and tooth number), the surfaces involved, the time needed for treatment, and the satisfaction of the patient with the treatment.

The treatment was provided by two previously trained and calibrated dentists in a mobile dental unit. Operators were trained on all steps of both protocols, including the pressure to be exerted with the polymer bur using a precision scale. Both protocols were trained on a total of 24 extracted teeth. Moreover, training was conducted clinically in four molars of two children that were not part of the study.

Preparation was similar for both protocols. To prepare the patients prior to the treatment, the management technique “tell-show-do”^17^ was applied. After cleaning the tooth with a rotatory brush, relative moisture control using cotton wool rolls and suction was performed. If needed, cavities were opened using water-cooled diamond burs (1012 e 1014, KG, Sorensen, Cotia, Brazil). Conventional stainless steel rose head burs (3 and 5, Maillefer, Dentsply, Konstanz, Germany) were used in low rotation to remove peripheral carious dentin until only hard, dry dentin remained. The removal of the pulp-axial carious dentin was performed differently in both arms:

- O: Objective, self-limiting polymer bur removal. The polymer bur (PolyBur) was used on low rotation until the bur abraded and further removal of softened dentin was not possible.
- S: Subjective removal using hand excavation. Removal of carious dentin was performed using hand instruments (Duflex, Rio de Janeiro, Brazil), and selective removal to leathery, slightly moist and reasonably soft dentin (i.e., dentin which cannot be removed using an excavator without force) was performed^1^.

Local anesthesia (Alphacaine 100, Nova DFL, Rio de Janeiro, Brazil) was applied only if the child reported pain during de procedure (only two cases in the O group). All cavities were restored with a glass hybrid restorative system (Equia Forte, GC, Tokyo, Japan) following the manufactures’ instructions as follows. The cavity was conditioned with the GC Cavity Conditioner for 10 seconds, then rinsed and the excess of water removed with a cotton pellet. After capsule activation and mixing, the material was inserted into the cavity, using the capsule applier. Digital compression, excess removal and occlusal checking were performed before the surface was coated with Equia Forte Coat, which was light cured for 20 seconds. On multi-surfaces cavities, the use of a metallic matrix was required.

Recording of treatment times was only started when the operator removed the first instrument from the tray; the assistant set the timer. The timing was completed when the operator stated that the Equia Forte Coat was finally cured. If several restorations were performed in the same patient, this time measurement was performed for each restoration.

Upon completion of each restoration, patients were asked by the assistant in the absence of the operator ‘how satisfied were you with this treatment?’. For this purpose, a Likert Scale was used, with five satisfaction points (very satisfied, satisfied, neutral, dissatisfied and very dissatisfied), illustrated by facial expression figures, with the purpose of facilitating the comprehension of the child.

### Follow-up

The restorations were evaluated by two independent trained evaluators, who did not take part in the treatment phase, using the ART criteria^18,19^. Calibration and training of the evaluators on the ART criteria was performed before the study in children that attended the pedodontics service of the University Dental Clinic until a good agreement was observed. Duplicate examinations during follow-up were performed in 15 children to enable Kappa calculation (inter-examiner agreement: 0.85) and repeated after 30 days (intra-examiner agreement: 0.87 for both examiners). Moisture control was performed using cotton rolls and continuous aspiration. The restorations were classified as ‘success’ if they were present and satisfactory, or if a slight marginal defect was observed (scores 00 and 01). Codes 2–6 featured restorative failures that could be arising from fracture or a secondary carious lesion. Restorations with codes 7–9 indicated censored information. Pulpal complications were determined via the assessment of pain, sensitivity to percussion or cold/hot, swelling, sinus formation or the resulting need for extraction. The evaluators were equipped with headlamps (Kudos, Hong Kong, China), dental mirrors and CPITN probes (Golgran, São Caetano do Sul, Brazil).

### Statistical analysis

Descriptive statistics and pairwise testing using t- and Chi-square test were applied. Time and patient satisfaction data were statistically analyzed by generalized linear mixed models, with the covariates protocol (S vs. O), dental arch (upper vs. lower), primary molar (first vs. second), cavity extension (single- versus multi-surfaced) and operators (1 vs. 2); the patient and the school were introduced as random factors. Restoration survival was evaluated using multi-level Cox-test, accounting for clustering. The confidence level was set at 95% (α = 0.05). The analyses were performed using SPSS 24 (IBM, Armonk, USA). Multiple imputation and sensitivity analyses are planned for the final evaluation but were not conducted for the present interim evaluation.

### Ethical approval and informed consent

The trial was approved by the Research Ethics Committee of the University of Brasília Medical School (1.400.687/2016) and registered at Clinicaltrials.gov (NCT02754466). Informed consent was obtained from all the parents/legally authorized representative of the participants included in the study. We confirm that all research was performed in accordance with relevant guidelines (Declaration of Helsinki) and regulations.

## Results

The sample characteristics are presented in Table 1. A total of 177 restorations were placed, 86 using the objective protocol (O) and 91 using the subjective protocol (S), in 115 children (55 O, 60 S). There were no significant differences (p > 0.05) between the patients in both arms with regards to patients’ age or sex, or dental arch, primary molar, or surface extension of the treated lesions.

**Table 1:** Characteristics of the sample.

		Objective	Subjective
N_patients_		55	60
Sex	Male	28 (51%)	27 (45%)
	Female	27 (49%)	33 (55%)
Mean (SD) age		8.15 (±0.52)	8.36 (±0.52)
d_3_mft (SD) at baseline		3.61 (±2.1)	4.08 (±2.4)
N_restorations_		86	91
Primary molar	First	38 (44%)	44 (48%)
	Second	48 (56%)	47 (52%)
Arch	Upper	32 (37%)	34 (37%)
	Lower	54 (63%)	57 (63%)
Cavity extension	Single-surface	31 (36%)	26 (29%)
	Multi-surfaces	55 (64%)	65 (71%)

There were no significant differences between groups (p > 0.05).

The mean (95% CI) treatment time (Table 2) was 412 (382–441) s and 433 (404–462) s in O and S, respectively (p > 0.05). Also, no significant differences in time according to molar, lesion extension or operator emerged (p > 0.05). Treatment time was significantly shorter in lower than upper molars (p = 0.004).

**Table 2:** Effect of covariates on treatment time (GLM).

Covariate		Number of Restorations	Mean (s)	95% CI (s)	p-value
Protocol	O	86	412	382–441	0.378
	S	91	433	404–462	
Primary molar	First	82	421	391–452	0.766
	Second	95	424	396–452	
Arch	Upper	66	455	420–491	0.004
	Lower	111	403	379–427	
Cavity extension	Single-surface	57	409	372–447	0.891
	Multi-surfaces	120	429	404–453	
Operator	1	103	425	398–452	0.867
	2	74	420	388–451	

Mean time (in seconds) and 95% confidence intervals (95% CI) as well as levels of significance between groups (p-values) are shown. O: objective, polymer bur-based removal; S – subjective removal.

Patients were generally highly satisfied with their treatment (Table 3), without significant differences between groups (p > 0.05).

**Table 3:** Effect of covariates on patients’ satisfaction (GLM).

Covariate		Number of Restorations	Median	Mean	95% CI	p-value
Protocol	O	86	1.00	1.42	1.25–1.59	0.152
	S	91	1.00	1.59	1.40–1.79	
Primary molar	First	82	1.00	1.59	1.37–1.80	0.260
	Second	95	1.00	1.44	1.28–1.60	
Arch	Upper	66	1.00	1.74	1.46–2.03	0.067
	Lower	111	1.00	1.37	1.25–1.49	
Cavity extension	Single-surface	57	1.00	1.51	1.30–1.72	0.964
	Multi-surfaces	120	1.00	1.51	1.34–1.67	
Operator	1	103	1.00	1.58	1.39–1.78	0.356
	2	74	1.00	1.41	1.24–1.57	

Median and mean satisfaction (from 1- very satisfied to 5 – very dissatisfied) and 95% confidence intervals (95% CI) as well as levels of significance between groups (p-values) are shown. No significant differences emerged. O: objective, polymer bur-based removal; S – subjective removal.

No pulp exposures occurred. Two patients, both in group O, reported pain during restorative treatment, with local anesthesia being provided.

After a mean (SD, range) 13 (2; 8–18) months, 90 restorations in S and 84 in O were evaluated. Most complications were restorative (Table 4). Pulpal complications occurred in 5 (6%) and 2 (2.2%) molars of O and S, respectively. In all cases where a pulpal complication occurred, a restorative failure was also present. There were more failures in multiple surface restorations than in single surface restorations in both groups. Risk of failure was not significantly associated with the removal protocol, age, sex, dental arch or tooth type (p > 0.05/Cox). The only significant association was found between surface extension and survival (Table 5).

**Table 4:** Restoration survival according to restorative protocol and number of restoration surfaces after 1 year (n = restoration with follow-up).

	Objective			Subjective		
	Surface			Surface		
	Total (n = 84)	Single (n = 31)	Multi (n = 53)	Total (n = 90)	Single (n = 25)	Multi (n = 65)
Pulp survival	79 (94%)	30 (96.8%)	49 (92.4%)	88 (97.8%)	25 (100%)	63 (96.9%)
Restoration survival	62 (73.8%)	27 (87%)	35 (66%)	64 (71.1%)	25 (100%)	39 (60%)
Total survival	62 (73.8%)	27 (87%)	35 (66%)	64 (71.1%)	25 (100%)	39 (60%)

**Table 5:** Effect of covariates on survival (Cox).

Covariate		HR (95% CI)	p-value
Protocol	O (ref.)		
	S	0.963 (0.536–1.761)	0.902
Gender	Male (ref.)		
	Female	1.095 (0.551–2.173)	0.796
Age (per year)		0.963 (0.495–1.875)	0.912
Primary molar	First (ref.)		
	Second	0.574 (0.312–1.028)	0.062
Arch	Upper (ref.)		
	Lower	1.006 (0.551–1.837)	0.983
Cavity extension	**Single-surface (ref.)**		
	**Multi-surfaces**	**4.597 (1.700–12.431)**	**0.003**

Mean hazard rate (HR) and 95% confidence interval (95% CI) as well as levels of significance between groups (p-values) are shown. Significant differences are highlighted in bold. O: objective, polymer bur-based removal; S – subjective removal.

If using ART criteria, the total loss of the restoration (code 6) was most frequent, followed by restoration fracture (code 3) and by margin defects (code 2). Details are presented in Table 6.

**Table 6:** Restorative failure according to ART criteria (n = number of restorations).

ART code	ART code description	Objective		Subjective	
		Surface		Surface	
		Single (n = 31)	Multi (n = 55)	Single (n = 26)	Multi (n = 65)
0	Present, satisfactory	22	19	19	20
1	Present, slightly deficiency at cavity margin of less than 0.5 mm*	3	6	0	3
2	Present, deficiency as cavity margin of 0.5 mm or more*	2	4	0	4
3	Present, fracture in restoration	0	4	0	9
4	Present, fracture in tooth	0	0	0	0
5	Present, overextension of approximal margin of 0.5 mm or more*	0	0	0	0
6	Not present, most or all of restoration missing	2	10	0	13
7	Not present, other restorative treatment performed	0	0	0	0
8	Not present, tooth is not present**	2	10	6	16
9	Unable to diagnose	0	0	0	0
Drop-outs		0	2	1	0
**Exfoliated teeth		2	10	6	16

*Assessed using the 0.5 mm ball-end CPITN probe.

## Discussion

The present study reports an interim analysis of a randomized trial comparing objective versus subjective carious tissue removal in deep carious lesions in primary molars. Using a removal method which determines when to stop removal could increase the uptake of selective removal among practitioners (who only slowly tend to adopt this technique), mainly as the endpoint of removal is more reliable. It may also allow standardizing removal for scientific purposes. We found that, after 1 year, no significant differences in success or survival occurred, with most failures being restorative. Generally, the annual failure rate was high. Treatment time was not statistically different between the two protocols, while patients rated both strategies as highly satisfying.

While there is only limited clinical data on self-limiting, objective carious tissue removal, it has been reported that such burs allow a reproducible level of selectivity^20,21^, while it remains unclear if this yields any advantage when compared with subjective selective removal. We assessed the success and survival in O and S groups. In the present study, the low number of pulp exposures that occurred for both groups may be related to the training provided to both operators and to the fact that operators could gauge the radiographic extend and lesion depth prior to the intervention. We also found the risk of failure to be similar in both groups, with annual failure rates being slightly higher than what was reported by other studies on primary teeth that also used a glass hybrid restorative system^22,23^. One plausible explanation may be that we only included deep and mainly extended cavities, which might have had a negative influence on restoration survival. Clearly, even using selective (instead of non-selective) removal, the management of deep lesions in primary molars with a direct restorative approach is challenging. Our study calls for a more biologically grounded approach, e.g. involving the Hall Technique or non-restorative cavity control (where lesions are not restored, but inactivated). The latter, however, has been found to come with limited efficacy in non-adherent groups^24^.

Overall, our study does not confirm that O is superior (which may be demanded given the possibly higher costs) but indicates that both strategies perform similar when it comes to pulpal and restorative complications. Hence, if desiring a more standardized, reproducible endpoint of removal, O may be justified. More long-term data are needed to confirm if different removal strategies truly do not come with different pulpal or restorative risks.

In terms of time, it has already been shown that primary molar cavity preparation with rotary burs tends to be faster than with manual instruments. However when the total time to complete the procedure (including placement of the restoration) is added, no significant difference remains^25^. Our data corroborate this assumption, as the total time spent for both O and S groups was not significantly different. However, it is important to highlight that the variance in treatment times was generally high, and our study was likely underpowered to detect significant differences. Given that the material costs for O (which are single-use instruments) are higher, it may well be that treatment costs are higher using O than S.

Procedures involving rotary instruments might induce anxiety in children during dental treatment^26^. We hence expected to detect a difference in the immediate patient satisfaction rates between O and S but could not confirm this assumption. It might be that applying the “tell-show-do” technique^17^ as part of the clinical routine to psychologically prepare patients^26,27^ might have facilitated favorable treatment conditions and patients’ satisfaction. Further, the treatment was performed at school and not a dental clinic, which may again be favorable for patients’ perceptions^26^.

In our study, multiple surface restorations were about five times more likely to fail compared to single-surface restorations, which is consistent with a large body of evidence^18,28,29^. Especially for such multi-surface cavities, the described biological approaches involving stainless steel crowns may be advantageous.

This study has a number of limitations. First, this is an interim analysis, and we cannot deduce longer term results. It should be highlighted that interim analyses may come with risks of erroneous conclusions due to repeated testing, but also limited robustness of the yielded data (given that the study was powered for the final follow-up). Notably, we decided to nevertheless conduct such analysis as our study may give guidance to practitioners who, at present, do not have any clinical evidence as to the efficacy of self-limiting burs. Also note that endpoints like pulp exposure, satisfaction or treatment time will not be affected by follow-up. As a caveat, further outcomes which we plan to assess during follow-up (like cost-effectiveness) could not be reported here. Second, this is a superiority study, while one might argue that for similar treatments like O vs. S, one could also conduct a non-inferiority trial, which comes with different concepts in sample size estimation and statistical power. We, however, strongly believe that the self-limiting excavation should prove superiority given that there is a valid, established, applicable and easy way to conduct selective removal using subjective criteria and a hand excavator or conventional bur. Even when considering alternative outcomes like treatment time or comfort, which may justify the use of the self-limiting excavation in case the clinical efficacy may not be significantly different from that of conventional excavation, the notion of superiority is upheld. Third, given that no radiographic follow-up was performed due to radiation protection, we are likely to underestimate the true risk of pulpal failures (e.g. inter-radicular lesions or resorptions might not have been detected). Fourth, we used the child’s satisfaction as patient-reported outcome, while admittedly, pain during treatment could have been measured, too. It is known that measuring pain in children is challenging, especially when the pain level is low, and that different scales do not necessarily agree with each other^30^. Last, dropouts were handled as missing at random, and no imputation was performed for the present interim analysis. We will consider more extensive handling of attrition in the final report, and generally assume the extremely low attrition in this analysis to not have a relevant impact on our findings.

## Conclusion

Within the limitations of this interim analysis, objective and subjective selective carious tissue removal did not present significantly different success and survival. Despite deep lesions being managed, the risk of pulpal complications was low, and most failures were restorative by nature (regardless of the study arm). Both treatment time and immediate patient satisfaction did also not differ significantly between protocols. As expected, multiple surface restorations had a higher failure rate than single surface restorations. It is important to emphasize that these data stem from a one-year interim evaluation, and that longer follow-up assessments may provide important additional information. Based on the outcomes of this analysis, though, dentists may consider both objective and subjective selective carious tissue removal for deep lesions in primary molars.

### Data availability

The database can be made available on request provided data protection rules can be fulfilled.

## Supplementary information

Supplementary information.
